# Restoring the youth of aged red blood cells and extending their lifespan in circulation by remodelling membrane sialic acid

**DOI:** 10.1111/jcmm.12721

**Published:** 2015-11-18

**Authors:** Yao‐Xiong Huang, Wei‐Wei Tuo, Di Wang, Li‐Li Kang, Xing‐Yao Chen, Man Luo

**Affiliations:** ^1^ Department of Biomedical EngineeringJi Nan UniversityGuang ZhouChina

**Keywords:** aged red blood cell, sialic acid remodelling, restoring youth, extending lifespan

## Abstract

Membrane sialic acid (SA) plays an important role in the survival of red blood cells (RBCs), the age‐related reduction in SA content negatively impacts both the structure and function of these cells. We have therefore suggested that remodelling the SA in the membrane of aged cells would help recover cellular functions characteristic of young RBCs. We developed an effective method for the re‐sialylation of aged RBCs by which the cells were incubated with SA in the presence of cytidine triphosphate (CTP) and α‐2,3‐sialytransferase. We found that RBCs could be re‐sialylated if they had available SA‐binding groups and after the re‐sialylation, aged RBCs could restore their membrane SA to the level in young RBCs. Once the membrane SA was restored, the aged RBCs showed recovery of their biophysical and biochemical properties to similar levels as in young RBCs. Their life span in circulation was also extended to twofold. Our findings indicate that remodelling membrane SA not only helps restore the youth of aged RBCs, but also helps recover injured RBCs.

## Introduction

Human red blood cells (RBCs) have a lifespan of approximately 120 days, during which, they travel approximately 240 km making 170,000 circuits through the heart to different tissues and organs. Circulating RBCs must constantly squeeze through narrow capillaries 1, 2, 3, producing ‘wear and tear’ during which surface sialoglycoproteins and sialic acids (SAs) are sheared off. The structures and properties of the cells therefore change, and the cells age. The amount of membrane SA in an old RBC (aged about 90–120 days) is approximately 30% less than that of a young cell(aged 1–30 days) 4. The deformability of the cell membrane, the oxygen‐carrying capacity, and the amounts of Na^+^, K^+^‐ATPase, Ca^++^Mg^++^‐ATPase, and 2,3‐diphosphoglycerate (2,3‐DPG) also decrease concurrently 5. Red blood cells unfit for circulation are recognized and phagocytized by the reticuloendothelial system. The mean survival time of old RBCs is usually not more than 30 days 6. In a freshly drawn RBC unit, old RBCs comprise ~16% of the total RBCs 5.

Regarding RBCs stored in a blood bank, the preservation solution acidifies during storage, and the membrane SA content of RBCs decreases faster than in circulation 3, 5. The membrane SA of young RBCs decreases by ~35% after 21 days of storage, resulting in a decline in the structural and functional properties of the cells to levels similar to those of senescent cells 5. Thus, accelerated ageing accompanies the loss of membrane SA.

In addition to the decrease in membrane SA seen in aged cells, when the membrane SA of RBCs is reduced experimentally by enzyme treatment, the biochemical and biophysical properties and survival time of the cells also suffer 7, 8. Similarly, since the effects of neuraminidases, significant fall in SA was noted as well on the RBCs of the patients with diseases such as diabetes mellitus, malaria, anaemia and inflammation like in sepsis 4, 9, 10, 11. So, the membrane SA of RBCs appears to correlate with cellular ageing, disease and survival time.

Sialic acid is an acidic nine‐carbon sugar typically found as the outermost unit of glycan chains on the surface of all vertebrate cells and on secreted glycoproteins 12, 13. They are also found in some invertebrates and in certain bacteria that interact with vertebrates 13, 14. Sialic acids of human RBCs are mainly N‐acetylneuraminic acids (NANA), they account for 74–94% of the negative surface charge of RBC membrane 15, 16. The charge carried by SAs and other ionizable chemical groups is one of the major physicochemical drivers of various molecular and cellular interactions that affect immune reactions, apoptosis, receptor function, growth, differentiation, and ageing 17, 18, 19, 20. It was proved that SAs govern the morphology, membrane deformability, oxygenation capacity, and even the structure and distribution of the intracellular haemoglobin (Hb) molecules in human RBC 4, 21, 22.

Given the importance of SAs in RBC structure and function and because SA content decreases with cell age, we have suggested that remodelling the SA in the membrane of aged cells would help recover cellular functions characteristic of young RBCs. To test our hypothesis, we incubated aged RBCs with SA in the presence of CTP and α‐2,3‐sialytransferase to induce restoration of membrane SA and then investigated if cell structure, function, and survival improved following re‐sialylation. The aged RBCs defined in here are: (*i*) the old human RBCs aged about 90–120 days in circulation. According to previous researches, they can be separated from freshly drawn venous blood with Percoll density gradient centrifugation as the dense cells in the bottom fraction 4, 5, 23, 24, 25 and designated as O cells. (*ii*) The RBCs from standard blood‐banked leukoreduced CPDA‐1 human RBC units stored for 21 days (designated B_21_ cells). (*iii*) The RBCs from rabbit venous blood stored for 21 days in blood‐bank conditions after drawing from the animal (designated A_21_ cells).

## Materials and methods

Red blood cells from freshly drawn venous blood were obtained from 42 young, healthy, non‐smoking adult volunteers (age 23–26 years, mostly laboratory personnel, providing written informed consent) and from 18 male rabbits (New Zealand white people). Our study protocol was approved by the Ji Nan University Animal Care and Use Committee and conformed to the Chinese Public Health Service Policy on Humane Care and Use of Laboratory Animals.

Heparinized venous blood was centrifuged three times at 2000 × g for 10 min. at 4°C with isotonic PBS (90 mM NaCl, 50 mM sodium phosphate, 5 mM KCl, 6 mM glucose, pH 7.4). Then, the washed RBCs were fractionated with Percoll centrifugation 4, 23. After centrifugation, about 5–10% of the light cells (termed young or Y cells) and the dense cells (termed old or O cells) were harvested separately, washed twice in PBS, and stored at 4°C until subsequent treatment and analyses 4.

Eight bags of standard blood‐banked leukoreduced CPDA‐1 RBC units were obtained from Guangzhou Blood Services Center, China and stored for 21 days. The RBCs of the units are designated B_21_ cells.

To incorporate SA, 1.0 ml diluted old RBCs or B_21_ RBCs was incubated with 100 μl NANA (Sigma‐Aldrich, China, 0, 20, 40, or 80 μg/ml), 0.5 ml CTP (7.4 mM; Sigma‐Aldrich), and 1.5 ml α‐2,3‐sialytransferase (0.18 U/ml; Sigma‐Aldrich, China).

The zeta potential of the cells was measured with a Zeta PALS potential analyser (Brookhaven Instrument Limited, USA) at 37°C. Sialic acids on the RBC membrane were labelled with fluorescein isothiocyanate (FITC)‐MAA (*Maackia amurensis*; Biological, USA) and the surface charge of RBCs was labelled with CdSe/ZnS core‐shell quantum dots (QDs; Wuhan Jiayuan QDs Company, Limited, China) as described previously 26.

Membrane bending modulus *K*_c_ was measured by the dynamic imaging and analysing method as previously reported 4, 27. Membrane deformability was also measured using a standard micropipette aspiration technique to mimic the situation of the cell travelling through capillaries in circulation 4.

Raman spectra of living erythrocytes were recorded with a JY RAM INV system using a 514.2‐nm excitation line from an Ar^+^ ion laser 28. The acquisition band was 600–1800 per cm with a spectrum resolution of 1 per cm. The Raman spectra were recorded for at least 18 erythrocytes each time. To study the distribution of Hb in a single erythrocyte, line‐mapping was performed with a scan step of 0.5 μm 23.

Adenosine triphosphate (ATP) enzymatic activity of blood cytolysates including that of both Na^+^, K^+^‐ATPase and Ca^++^Mg^++^‐ATPase in RBCs was determined by using an ultra‐micro ATPase assay kit (Jiancheng Bioengineering Institute, Nanjing, China), as was the level of 2,3‐DPG in RBCs (kit from EIAab Science Company, Wu Han, China).

To determine RBC survival, 18 New Zealand White rabbits (*Oryctolagus cuniculus*, provided by the Institute of Laboratory Animals, Jinan University) were randomly divided into three groups (six rabbits/group). Five millilitres of blood was taken from the ear artery of each rabbit and collected into a CPDA‐1–containing blood bag. Cells were washed (5000 × g, 8 min.) two times with normal saline (0.9% NaCl), producing leukoreduced RBC samples.

For the first group (G_1_), RBCs were incubated with 350 μg FITC (Sigma‐Aldrich, 70 μg/ml) for 30 min., and then washed (400 × g, 5 min.) three times with normal saline to eliminate residual FITC. The labelled RBCs were then re‐suspended to their original volume in normal saline and autologously transfused into the rabbits through the marginal ear veins. After re‐infusion of the labelled RBCs, blood samples (5 μl per rabbit) from the marginal ear vein were collected into heparin‐containing tubes after 1 and 24 hrs and then every other day. The samples were washed with normal saline (400 × g, 5 min.), suspended in 2 ml normal saline, and assayed with a flow cytometer (FACSCalibur; BD, USA) to determine the percentage of FITC‐labelled RBCs. The half‐life of the labelled RBCs was the number of days at which the survival rate was 50%.

For group 2 (G_2_) and group 3 (G_3_), the blood samples were stored for 21 days and then prepared as leukoreduced RBCs. The G_2_ RBCs were incubated with 2.5 ml SA (Sigma‐Aldrich, 40 μg/ml), 1.5 ml α‐2,3‐sialytransferase (Sigma‐Aldrich, 0.18 U/ml), and 0.5 ml CTP (7.4 mM) for 4 hrs. Then the cells were washed (700 × g, 5 min.) two times with normal saline to eliminate residual SA. Similarly, after labelling with FITC, the G_2_ and G_3_ RBCs, the latter of which were not incubated with SA, were re‐infused into the animals. The sampling and assay procedure were the same as that for G_1_ RBCs.

Statistical analyses were performed with *t*‐tests using SPSS 15.0 statistical software (IBM, Armonk, NY, USA). *P*‐values were obtained by comparison of cells without or with incorporation of SA.

## Results

### Remodelling of aged cells’ membrane SA

**Figure 1:**
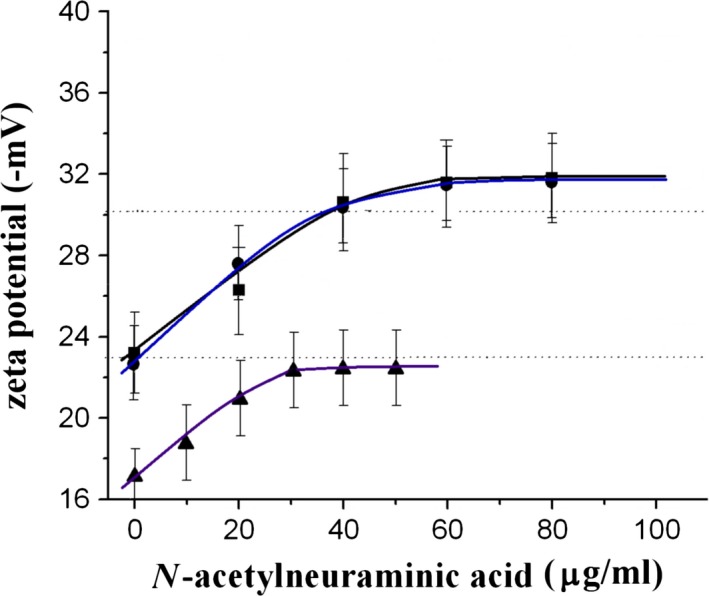
Membrane zeta potential of aged cells as a function of SA concentration. Solid squares: human O cells; solid circles: human B_21_ cells; solid triangles: A cells. The two dashed lines indicate the average zeta potential of human Y RBCs (upper line) and rabbit Y RBCs (lower line). The data and error bars are the mean ± SD.

**Figure 2:**
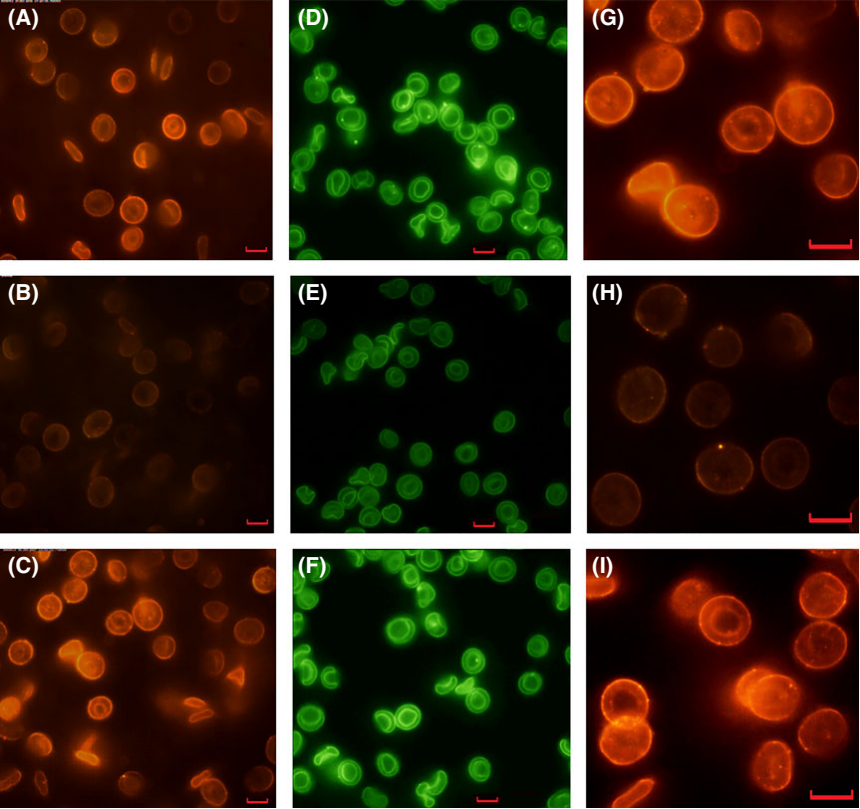
Fluorescence images of RBCs. Red fluorescence: QD‐labelled total surface charges; green fluorescence: FITC‐MAA–labelled membrane SAs. Scale bars in the images are 5 μm. (**A**) Y RBCs; (**B**) O RBCs; (**C**) SA‐restored O RBCs. (**D**) Y RBCs; (**E**) O RBCs; (**F**) SA‐restored O RBCs; (**G**) Y RBCs of freshly drawn blood; (**H**) B_21_ cells; (**I**) SA‐restored B_21_ cells.

From Figure 1 we can see that the zeta potential of aged cells increased with increasing SA concentration, and reached a value similar to that of young cells at ~40 μg/ml of supplemental SA but plateaued above the SA concentration. The membrane zeta potential is a marker of membrane SA, so Figure 1 suggests that aged RBCs could be effectively re‐sialylated with our method but the re‐sialylation would be saturated when the cells’ zeta potentials reached a value comparable to that of young cells. Similar phenomenon was also observed in rabbit RBCs as shown in the figure. Figure 2 shows simultaneously the content and distribution of SA (labelled with FITC‐MAA) and the surface charge (labelled with QDs) of the cells using a method described previously 26. The fluorescence intensity of the SA‐restored O cells indicated that both the FITC‐MAA–labelled membrane SAs and the QD‐labelled total surface charge had been restored to similar levels as in young cells. In addition, similar to young cells, both the membrane SA and the total surface charge were homogenously distributed on the membrane of the SA‐restored O cells. A similar result was obtained for SA‐restored B_21_ cells (Fig. 2I).

### Recovery of the cells’ morphological and rheological properties

As seen in Figure 2, the aged cells were more likely to be spherocyte, although echinocytes were also observed. The contact area (43.17 ± 2.16 μm^2^) and major axis (7.43 ± 0.22 μm) of O cells were significantly smaller (*P* < 0.05) than the values for young RBCs (46.42 ± 4.61 μm^2^ and 7.85 ± 0.55 μm respectively). Aggregation of O cells was also observed. After incubation with SA, however, the O cells assumed a discocyte shape similar to young cells and aggregation was no longer observed. Similarly, after incubation with SA (80 μg/ml), B_21_ cells recovered their morphology, *i.e*. changed from 40.07 ± 4.16 μm^2^ for contact area and 7.31 ± 0.65 μm for major axis to 44.09 ± 5.68 μm^2^ and 7.88 ± 0.54 μm, respectively.

**Figure 3:**
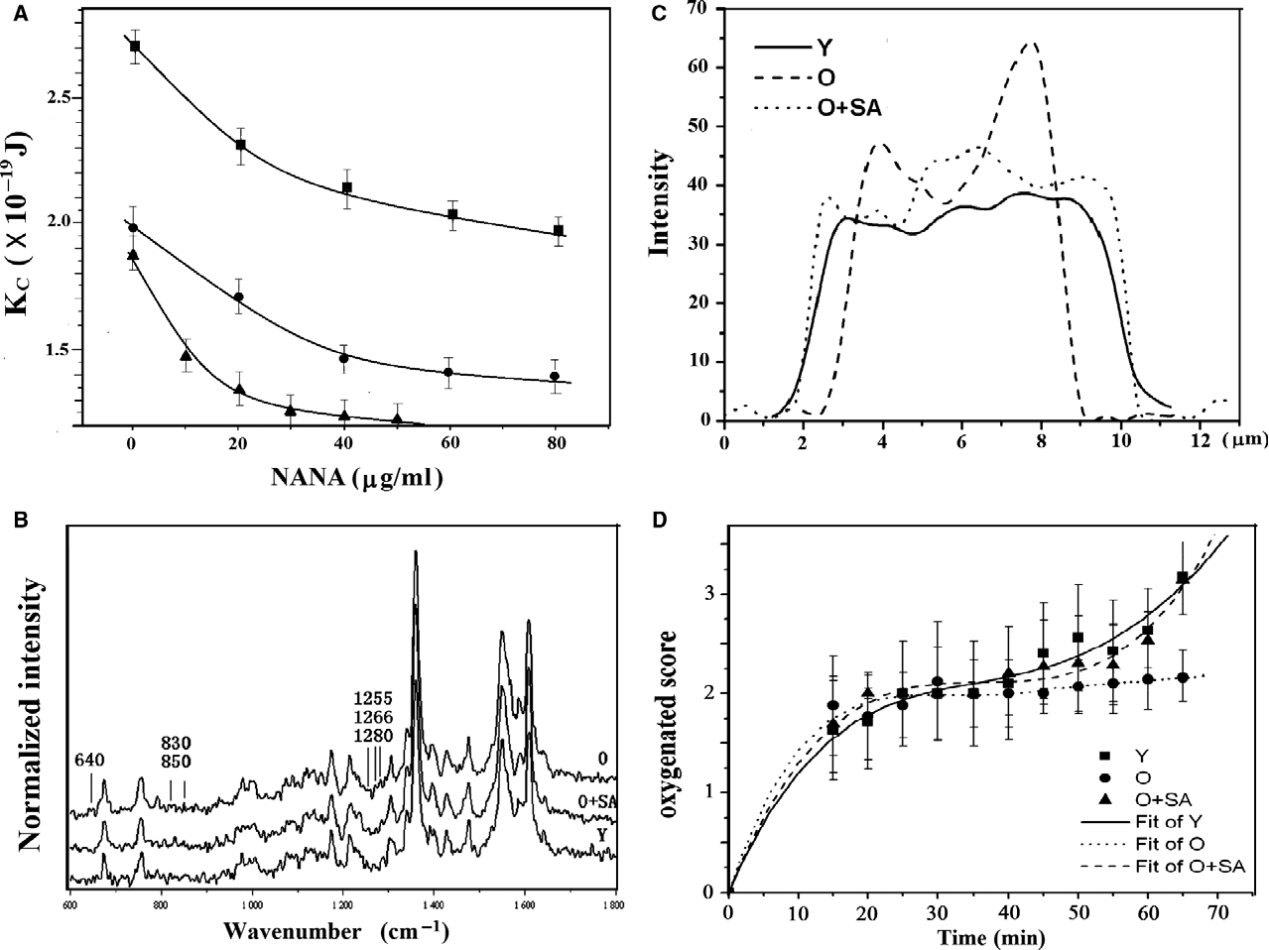
Biophysical properties of RBCs. The data points and error bars are the mean ± SD. (**A**) The bending modulus, K_c_, of RBCs as a function of SA concentration. Solid circles: O RBCs; solid squares: B_21_ RBCs; solid triangles: A RBCs. (**B**) Raman spectra averaged from 32 recorded spectra of each type of cells (O cells, SA‐restored O cells and Y RBCs). (**C**) Raman line mappings indicate the Hb distribution in O cells, Y RBCs, and SA‐restored O cells. (**D**) Transition from the deoxygenated (T) state to the oxygenated (R) state of O cells, Y RBCs, and SA‐restored O cells. The T, M1, M2, M3, M4 and R states are scored as 0, 1, 2, 3, 4, 5 respectively.

Figure 3A shows the bending modulus, *K*_c_, of the three types of cells (O, B_21_, and A cells) as a function of SA concentration. As described previously 4, *K*_c_ is inversely proportional to membrane deformability. The *K*_c_ of both O and B_21_ cells decreased with SA concentration and at 80 μg/ml the *K*_c_ was the same as that of young cells. To determine if the cells had recovered sufficient deformability to travel through capillaries in circulation, we aspirated the cells into a capillary with an inner diameter of 1.8 μm. The time needed to aspirate the SA‐restored cells into the capillary under a negative pressure of 1500 Pa was 0.66 sec., which was almost the same as that of young cells (0.67 sec.) but significantly shorter than the time for O cells (0.82 sec., *P* < 0.01). Similar results were obtained for re‐sialylated A cells.

### Changes in the structure and functions of intracellular protein

To determine if restoration of SA affected the O_2_‐binding capacity of Hb in RBCs, we performed confocal Raman micro‐spectroscopy on the cells. Figure 3B shows the average spectra of young, O and SA‐restored O RBCs. Most characteristic Raman bands of young, O and SA‐restored O RBCs were similar, but O cells showed more intense bands at 640, 830–850, 1266 and 1280 per cm. As reported previously 4, 28, 29, the bands at 830 and 850 per cm represent absorbance by Tyr. The ratio of I_830_/I_850_ in the Raman signal of old RBCs was less than 1, indicating that Tyr was exposed. The bands at 1266 and 1280 per cm represent the unordered coil and the α‐helix protein conformations of amide III respectively 30. Thus, the intense bands of the O cells suggest congregation and conformational changes in the side chains of Hb. After incorporation of SA, however, the bands became less intense (Fig. 3B), indicating that their Hb function was similar to that of young cells, and the SA‐restored aged RBCs improved their affinity of Hb for O_2_.

The distribution of intracellular Hb was also restored after incorporation of SA (Fig. 3C). In contrast to the homogeneous distribution in young cells, Hb intensity was much higher at the edge of O cells than in the centre region, indicating that more Hb was distributed around the cell membrane or bound to the membrane. This observation is consistent with results obtained previously 31.

To determine whether SA remodelling improved the O_2_‐carrying capacity of aged cells, we used Raman microscopy to measure the speed at which cells transitioned from a deoxygenated (T) state to an oxygenated (R) state *via* interrelated states that are named M1, M2, M3 and M4 28 respectively (Fig. 3D). The O cells required more time than young cells to become oxygenated from the T state. However, SA‐restored O cells showed a similar transition rate as the young cells, suggesting that their capacity to carry O_2_ had recovered.

### The viability of SA‐restored cells

**Figure 4:**
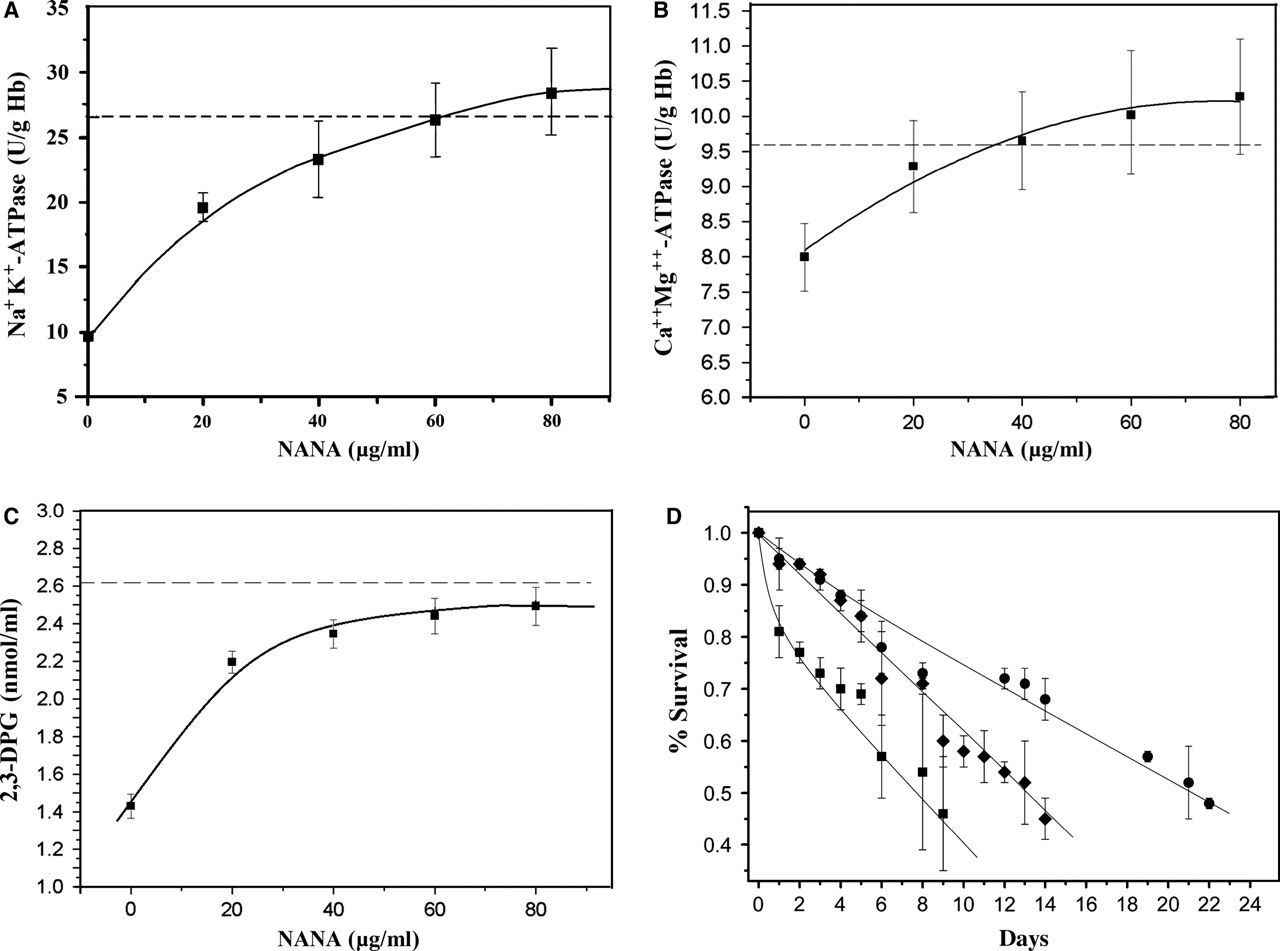
Biochemical properties of B_21_ cells as functions of SA concentration and the per cent survival of RBCs in circulation. (**A**) Na^+^, K^+^‐ATPase activity; (**B**) Ca^++^Mg^++^‐ATPase activity; (**C**) 2,3‐DPG level; (**D**) Percent survival of RBCs (solid circles: A cells from freshly drawn blood of rabbits; solid diamonds: SA‐restored A cells stored for 21 days; solid squares: A cells stored for 21 days. The dashed lines in (**A**–**C**) indicate the average level of each biochemical property for Y RBCs.

To investigate the relative viability of SA‐restored cells, we measured Na^+^, K^+^‐ATPase activity, Ca^++^Mg^++^‐ATPase activity and the 2,3‐DPG level of SA‐restored B_21_ cells. The activity of Na^+^, K^+^‐ATPase and Ca^++^Mg^++^‐ATPase and the 2,3‐DPG level in the aged cells increased with SA incorporation (Fig. 4A–C), indicating that the viability of the aged cells was improved after restoring membrane SA. On the other hand, the 2,3‐DPG level of RBCs reflects the affinity of Hb for O_2_, thus the restoration of the 2,3‐DPG level suggested that the efficiency of O_2_ unloading from Hb had been restored. These results are consistent with the Raman spectroscopy findings described above.

### Cell number of the SA‐restored RBC unit

According to our previous study 5, B_21_ RBC units can be just fractionated into three subpopulations with Percoll centrifugation. Young cells were not found in the unit and about 23% of the cells were lost compared with the freshly drawn blood sample. The remaining cells were senescent that were near death 5. However, the SA‐restored B_21_ cells can be fractionated into four subpopulations (young, M1, M2 and old cells) again by Percoll centrifugation, and the total number of cells was the same as in the freshly drawn blood sample unit 4. Even after storing the cells for 10 more days, the cell population could still be fractionated into four subpopulations without substantially changing the cellular properties. This suggests that the SA‐restored B_21_ cells can survive for longer time in blood‐bank condition.

### Life‐time of the SA‐restored RBCs in circulation

In the first group (G_1_) of animal experiments for RBC survival, RBCs from freshly drawn blood were labelled with FITC and immediately injected back into the animals. In the other two groups, RBCs were separated from animal blood that had been stored for 21 days; one group (G_2_) received cells that were incubated with SA (40 μg/ml), and the other group (G_3_) were not treated with SA. Approximately 94% of G_2_ cells survived after 24 hrs, which was similar to RBCs of G_1_ but significantly higher than cells that were stored for 21 days without SA incubation (G_3_, 80%, *P* < 0.01, Fig. 4D). In the first 8 days, the per cent survival of the SA‐restored aged RBCs was almost the same as that of the G_1_ RBCs, which had a half‐life of 14 days. Although the half‐life of G_2_ cells was shorter than that of G_1_ RBCs (22 days), the half‐life was significantly longer than G_3_ cells (8 days, *P* < 0.01), indicating that, after SA remodelling, the SA‐restored senescent cells showed improved viability and an extended lifespan.

### Biosafety and clinical potential of the SA‐restored aged RBCs

To evaluate the clinical potential of introducing SA‐restored aged RBCs into the circulation, we performed routine blood examination and health checks on animals in the three groups 2 days, 1 week and 2 weeks after RBC re‐infusion. All parameters of the rabbits were normal (data not shown). The animals in group G_2_ grew well, behaved normally, and were in good health at all times.

### Effect on unhealthy RBCs

To determine whether viability could be restored to unhealthy cells by treating them with SA, we performed an experiment with RBCs from patients with anaemia. Upon remodelling of SA (30 μg/ml), the zeta potential of the unhealthy cells increased from ¦−20.8¦ ± 0.8 mV to ¦−26.3¦ ± 0.6 mV, which was very close to the value of a control with normal RBCs (−27.1 ± 0.8 mV). Correspondingly, the *K*_c_ decreased from 1.85 ± 0.06 × 10^−19^ J to 1.68 ± 0.04 × 10^−19^ J (normal value: 1.62 ± 0.04 × 10^−19^ J), and about 32% of abnormally shaped cells returned to a normal discocyte shape. Thus, remodelling membrane SA also helped restore the structure and function of some unhealthy RBCs.

## Discussion

According to the results presented in Figures 1 and 2, we can see that by the aid of CTP and α‐2,3‐sialytransferase, SA can be effectively incorporated into the membranes of aged RBCs. The recovery of the RBCs’ zeta potential indicates that the cells indeed had been re‐sialylated because after incubation with SA, the RBCs were washed at least three times so no free SAs could be retained. This observation was confirmed by the presence of a dark background for both the FITC‐ and QD‐labelled samples (Fig. 2).

From Figure 1, we can also see that the zeta potential of the O cells increased with SA concentration but plateaued above 40 μg/ml, similar trends were seen in the curves of B_21_ cells and A_21_ cells. This suggests that the membrane SA only can be restored to a certain value and re‐sialylation requires unoccupied SA‐binding groups on the cell surface. To prove it, we incubated young RBCs from freshly drawn human blood with NANA. Although a very high concentration (≥80 μg/ml) of SA was used, the zeta potential of the cells increased just slightly from ¦−30.2¦ ± 1.2 mV to ¦−31.7¦ ± 0.8 mV and then plateaued as the behaviour of re‐sialylated‐aged cells shown in Figure 1. This experiment indicated that RBCs have a limited number of cell surface SA‐binding groups and that the membrane can be re‐sialylated only when such groups have not been occupied.

As demonstrated previously that SAs govern the morphology, membrane deformability, oxygenation capacity and even the structure and distribution of the intracellular Hb molecules in human RBC 4, 21, 22. The variation in the membrane SA and surface charge would cause collinear changes in these properties 4, 21, 22, 32. Therefore, once the membrane SAs have restored, all the properties are recovered. According to the findings of our present study and some other researches 4, 33, the recovery of both the properties is most likely mediated by the change in band 3 protein induced by the change in NANA carboxyls related‐charge. Band 3 is one of the key proteins influencing membrane flexibility and is also the major fraction of the Hb‐binding sites on RBC membranes 34. As aged RBCs lose some membrane SAs, they undergo membrane changes in band‐3 33, 35. The conformational change in band‐3 subsequently induces a series changes in the morphological and rheological properties of the cells 32, 36, 37. On the other hand, the decrease in SA/surface charge would induce changes of the conformation and aggregation as well as distribution of the intracellular Hb 4, 5, so that more intracellular Hb molecules would bind to the cytoplasmic domain of band 3 31. The binding of Hb to band 3 not only leads to reduced membrane flexibility 38, but also influences the propensity for Hb to become oxidized because the reactive oxygen species generated during autoxidation are not efficiently neutralized by cellular antioxidant enzymes when Hb is bound to the membrane 39, 40. Therefore, aged RBCs are less flexible and carry less oxygen. Upon restoration of membrane SA, however, less Hb was bound to the membrane, and its distribution was almost as homogeneous as that in young RBCs. The conformation of band 3 also restores. Thus, the flexibility of re‐sialylated RBCs was similar to that of young cells (Fig. 3A), and hence their oxygen‐carrying capacity also was expected to improve.

The recovery of the band 3 conformation after restoration of membrane SA in SA‐restored aged RBCs may be also a factor that helped restore the Na^+^, K^+^‐ATPase and Ca^++^Mg^++^‐ATPase activities and the 2,3‐DPG level because that recovery of band 3 conformation improves membrane permeability. Since membrane permeability correlates positively with ATPase activity/ATP concentration in RBCs, activation of the ion pump can significantly increase intracellular ATP 41.

## Conclusions

Our results show that remodelling membrane SA has a remarkable effect on aged RBCs, restoring their youth, extending their lifespan, and promoting recovery of cells that had been stored for a long time or that were dysfunctional. These findings have broad biological and medical implications for the recovery of aged and diseased cells and may be applicable to blood‐banked RBCs to prevent ‘storage lesion’, which was believed as one of the most critical issue facing transfusion medicine 42. Furthermore, RBCs are an ideal model for single‐cell investigation because the membrane is simple but exhibits functional activities that are representative of the plasma membrane of other cell types. Thus, our findings are important not only for revealing the correlation between membrane SA and RBC structure and function, but probably also help to establish ways to extend the lifespan of other cell types and treat diseased cells.

## Conflicts of interest

The authors have declared that no competing interests exist.

## Author contributions

YXH conceived and designed the experiments. WWT, DW, LLK, XYC performed the experiments. YXH and ML contributed analysing the data. YXH wrote the paper. YXH would like to thank Dr. J Mehrishi for the useful discussion on the effect of SA.

## References

[1] Adamson JW . New blood, old blood, or no blood? N Engl J Med. 2008; 358: 1295–6. 18354108 10.1056/NEJMe0800520

[2] Bosman GJ , Werre JM , Willekens FL , *et al* Erythrocyte ageing *in vivo* and *in vitro*: structural aspects and implications for transfusion. Transfus Med. 2008; 18: 335–47. 19140816 10.1111/j.1365-3148.2008.00892.x

[3] Chin‐Yee I , Arya N , d'Almeida M . The red cell storage lesion and its implication for transfusion. Transfus Sci. 1997; 18: 447–58. 10175158 10.1016/S0955-3886(97)00043-X

[4] Huang Y‐X , Wu Z‐J , Mehrishi J , *et al* Human red blood cell aging: correlative changes in surface charge and cell properties. J Cell Mol Med. 2011; 15: 2634–42. 21435169 10.1111/j.1582-4934.2011.01310.x PMC4373432

[5] Tuo W‐W , Wang D , Liang W‐J , *et al* How cell number and cellular properties of blood‐banked red blood cells of different cell ages decline during storage. PLoS One. 2014; 9: e105692 Doi:10.1371/journal.pone.0105692. 25167052 10.1371/journal.pone.0105692 PMC4148343

[6] Piomelli S , Seaman C , Reibman J , *et al* Separation of younger red cells with improved survival *in vivo*: an approach to chronic transfusion therapy. Proc Natl Acad Sci USA. 1978; 75: 3474–8. 277949 10.1073/pnas.75.7.3474 PMC392800

[7] Marikovsky Y , Elazar E , Danon D . Rabbit erythrocyte survival following diminished sialic acid and ATP depletion. Mech Ageing Dev. 1977; 6: 233–40. 865145 10.1016/0047-6374(77)90024-0

[8] Yao CC , Yao P , Huang YX . Influence of trypsin on the dynamic mechanical properties of intact RBC membrane. Chinese J Pathophysiol. 2007; 23: 991–4.

[9] Vahalkar GS , Haldankar VA . RBC membrane composition in insulin dependent diabetes mellitus in context of oxidative stress. Indian J Clin Biochem. 2008; 23: 223–6. 23105758 10.1007/s12291-008-0050-2 PMC3453436

[10] Bratosin D , Palii C , Moicean AD , *et al* Reduced diversity of the human erythrocyte membrane sialic acids in polycythemia vera. Absence of N ‐glycolylneuraminic acid and characterisation of N ‐acetylneuraminic acid 1,7 lactone. Biochimie. 2007; 89: 355–9. 17188794 10.1016/j.biochi.2006.11.004

[11] Varki A . Sialic acids in human health and disease. Trends Mol Med. 2008; 14: 351–60. 18606570 10.1016/j.molmed.2008.06.002 PMC2553044

[12] Traving C , Schauer R . Structure, function and metabolism of sialic acids. Cell Mol Life Sci. 1998; 54: 1330–49. 9893709 10.1007/s000180050258 PMC7082800

[13] Angata T , Varki A . Chemical diversity in the sialic acids and related α‐keto acids: an evolutionary perspective. Chem Rev. 2002; 102: 439–70. 11841250 10.1021/cr000407m

[14] Schauer R . Sialic acids: fascinating sugars in higher animals and man. Zoology. 2004; 107: 49–64. 16351927 10.1016/j.zool.2003.10.002

[15] Cook G , Heard D , Seaman G . Sialic acids and the electrokinetic charge of human erythrocytes. Nature. 1961; 191: 44–7. 13695245 10.1038/191044a0

[16] Eylar EH , Madoff MA , Brody OV , *et al* The contribution of sialic acid to the surface charge of the erythrocyte. J Biol Chem. 1962; 237: 1992–2000. 13891108

[17] Schauer R . Sialic acids as regulators of molecular and cellular interactions. Curr Opin Struct Biol. 2009; 19: 1–8. 19699080 10.1016/j.sbi.2009.06.003 PMC7127376

[18] Mehrishi JN . Molecular aspects of the mammalian cell surface. In: Butler JAV, Noble D, editors. Oxford: Pergamon Press; 1972. 10.1016/0079-6107(72)90013-2 4122510

[19] Seaman GVF . Electrokinetic behavior of red cells In: Surgenor DMacN, editor. The red blood cell. 2nd ed New York: Academic Press; 1975.

[20] Abramson HA . The cataphoretic velocity of mammalian red blood cells. J Gen Physio. 1929; 12: 711–25. 10.1085/jgp.12.6.711 PMC2323749 19872493

[21] Durocher JR , Payne RC , Conrad ME . Role of sialic acid in erythrocyte survival. Blood. 1975; 45: 11–20. 803103

[22] Bratosin D , Mazurier J , Tissier J , *et al* Cellular and molecular mechanisms of senescent erythrocyte phagocyosis by macrophages. A review. Biochimie. 1998; 80: 173–95. 9587675 10.1016/s0300-9084(98)80024-2

[23] Murphy GJ . Influence of temperature and method of centrifugation on the separation of erythrocytes. JR J Lab Clin Med. 1973; 82: 334–41. 4721385

[24] Lutz HU , Stammler P , Fasler S , *et al* Density separation of human red blood cells on self forming Percoll gradients: correlation with cell age. Biochim Biophys Acta. 1992; 1116: 1–10. 1371700 10.1016/0304-4165(92)90120-j

[25] Mueller T , Jackson C , Dockter M , *et al* Membrane skeletal alterations during *in vivo* mouse red cell aging. Increase in the band 4.1a:4.1b ratio. J Clin Invest. 1987; 79: 492–9. 3805278 10.1172/JCI112839 PMC424112

[26] Huang Y‐X , Zheng X‐J , Kang L‐L , *et al* Quantum dots as a sensor for quantitative visualization of surface charges on single living cells with nano‐scale resolution. Biosens Bioelectron. 2011; 26: 2114–8. 21111603 10.1016/j.bios.2010.09.016

[27] Li J , Huang Y‐X . Superresolution measurement on the minute fluctuation of cell membrane. Chin Sci Bull. 2006; 51: 143–7.

[28] Kang LL , Huang YX , Liu WJ , *et al* Confocal Raman microscopy on single living young and old erythrocytes. Biopolymers. 2008; 89: 951–9. 18615496 10.1002/bip.21042

[29] Huang Y‐X , Wu Z‐J , Huang B‐T , *et al* Pathway and mechanism of pH dependent human hemoglobin tetramer‐dimer‐monomer dissociations. PLoS One. 2013; 8: e81708. 24312337 10.1371/journal.pone.0081708 PMC3842943

[30] Bandekar J , Krimm S . Vibrational analysis of peptides, polypeptides, and proteins. VI. Assignment of b‐turn Modes in Insulin and other Proteins. Biopolymers. 1980; 19: 31–6. 6989414 10.1002/bip.1980.360190103

[31] Demehin AA , Abugo OO , Jayakumar R . Binding of hemoglobin to red cell membranes with eosin‐5‐maleimide‐labeled band3: analysis of entrifugation and fluorescence lifetime data. Biochemistry. 2002; 41: 8630–7. 12093280 10.1021/bi012007e PMC6980380

[32] Chen XY , Huang YX , Liu WJ , *et al* Membrane surface charge and morphological and mechanical properties of young and old erythrocytes. Current Appl Phys. 2007; 7: e94–6.

[33] Lutz HU , Bogdanova A . Mechanisms tagging senescent red blood cells for clearance in healthy humans. Front Physiol. 2013; 4: 1–15. 24399969 10.3389/fphys.2013.00387 PMC3872327

[34] Wang DN . Band 3 protein: structure, flexibility and function. FEBS Lett. 1994; 346: 26–31. 8206153 10.1016/0014-5793(94)00468-4

[35] Lutz HU , Stringaro‐Wipf G . Senescent red cell‐bound IgG is attached to band 3 protein. Biochim Biophys Acta. 1983; 42: 11–2. 6675681

[36] Gimsa J , Ried C . Do band 3 protein conformational changes mediate shape changes of human erythrocytes. Mol Membr Biol. 1995; 12: 247–54. 8520625 10.3109/09687689509072424

[37] Rudenko SV . Erythrocyte morphological states, phases, transitions and trajectories. Biochim Biophys Acta. 2010; 1798: 1767–78. 20538541 10.1016/j.bbamem.2010.05.010

[38] Salhany J , Cassoly R . Kinetics of p‐mercuribenzoate binding to sulfhydryl groups on the isolated cytoplasmic fragment of band 3 protein. Effect of hemoglobin binding on the conformation. J Biol Chem. 1989; 264: 1399–404. 2912963

[39] Rifkind J , Zhang L , Heim J , *et al* The role of hemoglobin in generating oxyradicals. Basic Life Sci. 1988; 49: 157–62. 2854980 10.1007/978-1-4684-5568-7_23

[40] Rifkind JM , Zhang L , Levy A , *et al* The hypoxic stress on erythrocytes associated with superoxide formation. Free Radical Res Commun. 1991; 12: 645–52. 1648015 10.3109/10715769109145842

[41] Ataullakhanov FI , Vitvitsky VM . What determines the intracellular ATP concentration. Biosci Rep. 2002; 22: 501–10. 12635847 10.1023/a:1022069718709

[42] Ness PM . Does transfusion of stored red blood cells cause clinically important adverse effects? A critical question in search of an answer and a plan. Transfusion. 2011; 51: 666–7. 21496035 10.1111/j.1537-2995.2011.03121.x

